# Expression patterns of *Phf5a/PHF5A* and *Gja1/GJA1* in rat and human endometrial cancer

**DOI:** 10.1186/1475-2867-13-43

**Published:** 2013-05-15

**Authors:** Eva Falck, Karin Klinga-Levan

**Affiliations:** 1 Systems Biology Research Centre – Tumor biology, School of Life Sciences, University of Skövde, Skövde SE-54128, Sweden; 2 School of Health and Medical Sciences, Örebro University, Örebro, Sweden

**Keywords:** BDII, Endometrial cancer, Genetic background, *Phf5a*, *Gja1*

## Abstract

Endometrial adenocarcinoma is the most frequently diagnosed cancer of the female genital tract in the western world. Studies of complex diseases can be difficult to perform on human tumor samples due to the high genetic heterogeneity in human. The use of rat models is preferable since rat has similarities in pathogenesis and histopathological properties to that of human.

A genomic region including the highly conserved *Phf5a* gene associated to development of EAC has previously been identified in an association study. PHF5A has been suggested to acts as a transcription factor or cofactor in the up regulation of expression of *Gja1* gene in the presence of estrogen. It has earlier been shown that the *Phf5a* gene is down regulated in rat EAC derived cell lines by means of expression microarrays.

We analyzed the expression of *Phf5a* and *Gja1* by qPCR, and potential relations between the two genes in EAC tumors and non-malignant cell lines derived from the BDII rat model. In addition, the expression pattern of these genes was compared in rat and human EAC tumor samples.

Changes in expression for *Phf5a/PHF5A* were found in tumors from both rat and human even though the observed pattern was not completely consistent between the two species. By separating rat EAC cell lines according to the genetic background, a significant lower expression of *Phf5a* in one of the two cross backgrounds was revealed, but not for the other. In contrast to other studies, *Phf5a/PHF5A* regulation of *Gja1/GJA1* was not revealed in this study.

## Background

Endometrial carcinoma arises from the endometrium, the inner lining of the uterus. Endometrial adenocarcinoma (EAC), the predominant sub type, is the most frequently diagnosed cancer of the female genital tract ranking fourth among the invasive tumors that affect women in the western world. Approximately 85% of the patients with the diagnosis EAC are over 50 years of age [1]. As most cancers, EAC is a complex disease and development of the tumors is influenced by multiple genetic alterations. As the endometrium is a hormone-dependent tissue, tumors developed in this tissue, including EACs, are mainly hormone-dependent [2]. It has been suggested that excess administration of estrogen may act as one of the main factors in predisposition to EAC in women [3].

Studies of complex diseases can be difficult to perform on human tumor samples due to the high genetic heterogeneity in human. Therefore, in studies of complex disease and as a complement to studies in human, model organisms such as inbred rat strains are often used. The use of rat models is preferable since rat has similarities in pathogenesis and histopathological properties to those of human [2,4]. In the EAC susceptible BDII rat strain, more than 90% of the virgin females develop tumors spontaneously during their lifetime. This tumor model has been genetically well characterized, but there is still much important genetic information that remains to be fully understood in this model [5].

Associations between certain marker alleles and tumor incidence in cross progenies, including the susceptible BDII strain and non-susceptible strains, were identified by means of genome-wide screening with microsatellites. It was clear from the data on tumors developed in the inter-strain crosses that the onset of tumors depends not only on the presence of susceptibility alleles from the EAC-prone BDII strain, but is also affected by the contribution from the non-susceptible strains [6,7].

In another study of the same tumor material developed in the inter-strain crosses expression profiling of tumor and pre malignant cell lines was performed. A number of genes were significantly differentially expressed between EAC and pre/non malignant cell lines, and several of these belonged to cancer-associated pathways [8]. One of the genomic regions associated to development of EAC identified in the association studies included *Phf5a,* and gene expression profiling analysis revealed this gene was down regulated in EAC derived cell lines.

The PHD finger-like domain protein 5a (PHF5A) is ubiquitously expressed and is located in the nucleus. The gene *Phf5a* and its human counterpart *PHF5A* (former name *Ini)* are located on rat chromosome 7 (RNO7q34) and human chromosome 22 (HSA2213q2), respectively. The rat gene consists of four exons, while the human gene have five exons. Both genes encode a highly conserved protein of 110 amino acids that contains a PHD finger domain. The PHD-finger domain is composed by eight amino acids and has been found in two major groups of proteins. One group consists of transcriptional activators, repressors and cofactors, and the second major group consists of proteins involved in chromatin modulating complexes such as acetyltransferase or complexes containing acetyltransferase [9].

The alignment between the coding sequence of human *PHF5a* to mouse *Phf5a* and rat *Phf5a* revealed a sequence identity of 91% and 94%, respectively and at the protein level the amino acid sequences are 100% identical between the three species [10].

PHF5A acts as a transcription factor or cofactor in the expression of the gap junction alpha 1 (*Gja1*) gene, which is normally up regulated in uterus. The PHF5A protein binds to the proximal region of the *Gja1* promoter and promotes the up regulation of *Gja1* by estrogen [9,11]. The *Gja1* gene encodes a Gap junction alpha 1 (former name connexin43) protein that is one of 21 different isoforms that belongs to the connexin family. Connexins are membrane proteins that form channels between adjacent cells and mediate cell-to-cell communication [12-15]. *Gja1* is expressed in various tissues like the brain, heart, ovary, uterus and smooth mussels including the myometrium. In the uterus, gap junctions are required for coordination of the contractions at the end of the pregnancy. In the myometrium *Gja1* is under control of a steroid hormone as being up regulated by estrogen and down regulated by progesterone [11].

The expression of *GJA1* is often down regulated in mammary carcinoma cell lines indicating that in these cases the role of GJA1 in carcinogenesis in maintaining cell differentiation and preventing transformation into cancer cells can not be fulfilled [16,17]. In addition it was found that the expression of *GJA1* decreases with increased grade of endometrial adenocarcinoma [18].

The aim of this study was to investigate the expression of *Phf5a* and its effect on *Gja1* expression in tumor and non-malignant cell lines derived from the BDII rat model with different genetic backgrounds. In addition, the expression pattern of these genes was compared to the expression pattern of the corresponding genes in the human tumor samples of FIGO grades I-III.

## Results

The gene expression of *Phf5a* and *Gja1* in the rat samples was measured in four groups defined by their cross origin (BDIIxBN)xBDII or (BDIIxSPRD)xBDII and cell type (EAC or NME). Five EACs and 5 NMEs with the BN background and 6 EACs and 6 NMEs with the SPRD background were analysed (Table 1). Gene expression of *PHF5A* and *GJA1* in the human material was measured in 30 human EACs in FIGO grade I-III (10 tumors from each grade), and 26 benign (12 secretory phase and 14 proliferative phase) were analyzed with the students t-test for differences between groups (Table 2).

**Table 1:** Overview of the rat tumor material used in this study

**Tumor**	**Background**	**Pathology**
NUT43	(BDIIxBN)xBDII	EAC
NUT50	(BDIIxBN)xBDII	EAC
NUT81	(BDIIxBN)xBDII	EAC
NUT97	(BDIIxBN)xBDII	EAC
NUT128	(BDIIxBN)xBDII	EAC
NUT75	(BDIIxBN)xBDII	NME
NUT110	(BDIIxBN)xBDII	NME
NUT118	(BDIIxBN)xBDII	NME
NUT122	(BDIIxBN)xBDII	NME
NUT129	(BDIIxBN)xBDII	NME
NUT7	(BDIIxSPRD)xBDII	EAC
NUT12	(BDIIxSPRD)xBDII	EAC
NUT41	(BDIIxSPRD)xBDII	EAC
NUT42	(BDIIxSPRD)xBDII	EAC
NUT47	(BDIIxSPRD)xBDII	EAC
NUT84	(BDIIxSPRD)xBDII	EAC
NUT48	(BDIIxSPRD)xBDII	NME
NUT56	(BDIIxSPRD)xBDII	NME
NUT58	(BDIIxSPRD)xBDII	NME
NUT68	(BDIIxSPRD)xBDII	NME
NUT89	(BDIIxSPRD)xBDII	NME
NUT91	(BDIIxSPRD)xBDII	NME
REF	Rat Embryo Fibroblast	

**Table 2:** Overview of the human tumor material used in this study

**Sample**	**Tumor grade**	**Tissue**
Endometrium		Normal
14	Proliferative phase	Benign
12	Secretory phase	Benign
10	Type I	Malignant
10	Type II	Malignant
10	Type III	Malignant

Prior to the statistical analysis of the Ct values, One-Way ANOVA were conducted for analyses of differences among replicates in the rat as well as the human material. There were no significant differences detected among the replicates in either of the data sets. Thus, the average Ct values of the replicates were used in the following calculations of the relative quantitative gene expression. Any undetected Ct values for either of the genes were set to the Ct value of 40 cycles, which corresponds to expression at very low levels.

Pearson´s correlation test on log 2-fold change was performed to explore potential correlation between *Phf5a/PHF5A* and *Gja1/GJA1.* No expression correlation between the two genes could be seen, in the rat or human material.

In the rat tumor set, comparison of the gene expression of *Phf5a* and *Gja1* between the EAC and NME samples showed a slight, but not significant, decrease in in expression of *Phf5a* in the EAC cell lines. The corresponding test for *Gja1* revealed no significant differences. In comparisons between all samples of the BN cross origin and the SPRD cross origin, no significant differences were detected for any of the genes investigated (Table 3)*.*

**Table 3:** **The P-values obtained from the independent sample *t*-test for differences between groups**

**Rat**		***Phf5a***	***Gja1***
EAC	NME	0.056	0.915
BN	SPRD	0.376	0.784
BN/EAC	SPRD/EAC	0.038^*^	0.528
BN/NME	SPRD/NME	0.333	0.935
BN/EAC	BN/NME	0.006^**^	0.606
SPRD/EAC	SPRD/NME	0.991	0.727
Human		***PHF5A***	***GJA1***
Type I-III		0.262	0.446^1^
Prol	Secr	0.685	0.156
Type I-III	Prol/Secr	0.000^***^	0.282
Type I	Prol/Secr	0.002^**^	0.559
Type II	Prol/Secr	0.000^***^	0.517
Type III	Prol/Secr	0.004^*cp^	0.081

* P< 0.05.

** P< 0.01.

***P< 0.001.

**Figure 1:**
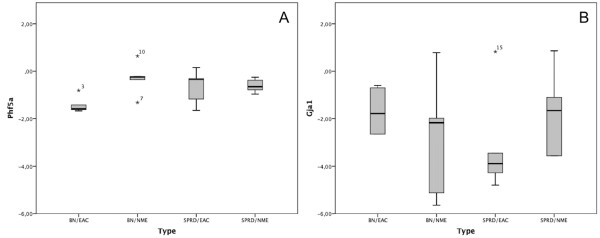
**Gene expression of *Phf5a*** **(A) and *Gja1*** **(B) in NME and NUT rat cell lines.** Gene expression of (**A**) *Phf5a* and (**B**) *Gja1* in rat endometrial adenocarcinoma and pre-malignant endometrial cell lines. The bars represent the mean delta delta Ct value in each group. The median in each group is represented by horizontal line.

When we performed the analysis separating groups by tissue type, EAC and NME, and cross background, (BDIIxSPRD)xBDII and (BDIIxBN)xBDII, still no significant differences were detected for *Gja1* in any of the comparisons. For *Phf5a* significant differences were found in comparisons between EACs developed in the BN and those in the SPRD background with a lower expression in the tumor cell lines derived from the progenies of the (BDIIxBN)xBDII crosses (P<0.05). The EAC cell lines developed in the (BDIIxBN)xBDII progeny displayed a significant decrease in *Phf5a* expression (P<0.01) when compared to the NMEs with the same background (Figure 1A and Table 3).

**Figure 2:**
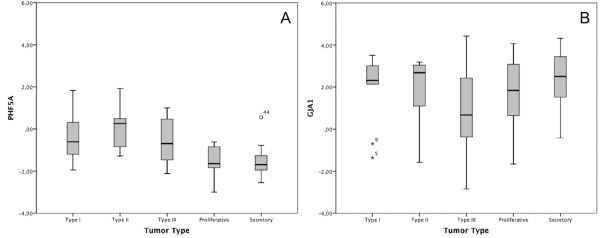
**Gene expression of *PHF5A*** **(A) and** ***GJA1*** **(B) in samples of FIGO grade I-III and benign samples.** (**A**) *PHF5A* and (**B**) *GJA1* gene expression in human endometrial adenocarcinomas of benign samples and FIGO grade I-III tumor samples. The bars represent the mean delta delta Ct value in each group. A horizontal line represents the median in each group.

In the human material no significant differences (P>0,05) between the proliferative and the secretory phase of the benign tumors for either *PHF5A* or *GJA1* were seen*,* and accordingly the two benign classes were merged (Table 3). An ANOVA test on the tumor classes revealed no significant differences among the different tumor grades (P>0,05, but still we analyzed the classes separately. For the gene *GJA1* no significant difference in any of the comparisons between malignant and benign material were detected (Table 2, Figure 2B). For *PHF5A* all tumor classes were up regulated compared to the benign samples, where type II tumors differed most from the benign samples (Table 3, Figure 2A).

## Discussion

From the results of the association studies in the BDII model of EAC, one small genomic region associated to the development of EAC that included the *Phf5a* gene was identified [6,7]. In an expression profiling study, the *Phf5a* gene was shown to be down regulated in certain EAC cell lines from tumors developed in the F2 and N1 progenies [8]. The chromosomal localization of *Phf5a* is RNO7, band q34, and the *PHF5A* corresponding human region is located on HSA22 (HSA2213q2), and the gene is highly conserved through evolution.

It has been suggested that the PHF5A protein plays a complex role as a general transcriptional activator for different genes. Trappe et al. (2002) suggested the PHF5A yeast orthologue plays a crucial roll for cell viability and survival [9]. In *C. elegans* the Phf5a orthologue displays a tissue- and stage-specific pattern of expression during morphogenetic development [19]. In rat myometrium the phf5a protein has been suggested to function as a transcription factor for the gene *Gja1* in the presence of estrogen by binding to the proximal promoter region and enhance expression of *Gja1*[11]. The protein encoded from the gene *Gja1* is a component of the gap junctions that form intercellular channels for diffusion of molecules between cells. It has been suggested that *GJA1* is down regulated in different human cancer types, presumably through promoter hyper-methylation, and that it displays tumor suppressor activity [17,20]. However, down regulation of GJA1 through its promoter hyper-methylation was shown not to be true for in human colorectal cancer [20,21], and therefore silencing of the *GJA1* was suggested be mediated by other mechanisms through estrogen activation. One possible mechanism could be up regulation of *Gja1* by *Phf5a* through the action of estrogen as suggested by Oltra et al. [11].

In this study, we investigated the expression*,* and a potential correlation of *Phf5a* and *Gja1* in rat cell lines. As the cell lines were derived from tumors developed in crosses between the females of the BDII inbred strain, susceptible to develop EAC and two non-susceptible strains (BN and SPRD), the impact of the genetic background could also be taken into consideration. In human the influence of the genetic background on the development and path of tumourigenesis is difficult to grasp in human clinical materials. Still, there are some cases were specific alleles have a protective or enhancing effect on the expression of mutation alleles in *BRCA1/BRCA2*[22,23]. In inbred animal models there are good opportunities to design crosses and experiments to enable studies of the impact of the genetic background on the outcome of carcinogenesis. The BDII rat model has been used for studies of EAC onset and development on the DNA, as well as the genomic level. In association studies, it was proved that depending on cross background, (BDIIxSPRD)xBDII and (BDIIxBN)xBDII, different genomic regions were associated to the onset of tumors [7,24]. SKY analysis of genomic aberrations in the cell lines derived from the tumors developed among females in the cross progenies, revealed that some aberrations in the genome were common to both cross backgrounds and others occurred only in one of the cross backgrounds [25].

Without considering the genetic background of rat female progenies that developed tumors, the expression of *Phf5a* in EAC cell lines was shown to be slightly lower than the NME samples, but not significantly lower. By separating the cell lines according to cross background, a significant lower expression of *Phf5a* in the EAC derived cell lines with the BN background compared to the EACs from the SPRD background was revealed. *Phf5a* was not differentially expressed in comparison with the non-malignant cell lines in the SPRD background, but in the BN background (Figure 1A). Accordingly, cross set-ups such as in the BDII rat model, permit findings that otherwise is difficult to uncover.

The normal function of *GJA1/Gja1* is in the myometrium is coordination of the contractions at the end of the pregnancy, and is under control of a steroid hormone Expression of *GJA1* has been shown to be down regulated in mammary tumors, lung cancer and endometrial adenocarcinoma [18,26-29], as *GJA1/Gja1* normally maintains cell differentiation and prevents transformation into cancer cells [11,16,17]. In addition it has been found that the expression of *GJA1* decreases with increased grade of endometrial adenocarcinoma [18].

This earlier results could not be validated in this study as the *Gja1* gene was not down regulated in the rat EAC cell lines, and no significant differences between the different cross backgrounds or between EACs and NMEs were detected. It is known that the PHF5A protein binds to the proximal region of the *Gja1* promoter and promotes the up regulation of *Gja1* by estrogens as described in transfections experiments [9,11]. In these experiments, it was stated that PHF5A was localized in the nucleus, where it binds to the *GJA1* promoter and up regulates the expression of *GJA1* in the presence of estrogens in a tissue specific and dose dependent way. In contrast to the studies describes above [11], it could not be corroborated that Phf5a regulate the expression of *Gja1* in experimental EACs as no correlation between the expression of *Gja1* and *Phf5a* could be seen (Table 3, Figure 1B).

The result of the expression study of *Phf5a* in rat could not be verified in the human samples. Although *PHF5A* was also down regulated in the human tumor samples, but unexpectedly, it was more down regulated in the human benign samples.

## Conclusions

To conclude, changes in expression in tumors for one of the genes, *PHF5A/Phf5a* were found both in human and rat, even if the pattern of changes is not completely consistent between the two species. The reason for this discrepancy has to be further investigated. We could not confirm suggestions made in earlier studies, where it was shown that PHF5A regulate the expression of GJA1. The impact of estrogen in this regulation has been investigated in the same study [11]. We plan to investigate and validate this in the material used in an extended study, where we will also investigate the function of PHF5A as a promoter for other genes.

The utmost important finding in this study was that, by using an experimental model, we could successfully show that certain aberrations in the tumors that lead to changes in gene expression, and subsequent changes in protein expression is dependent on the genetic background. Thus, the importance of using animal models as a complement to clinical studies is obvious.

## Material and methods

### Rat tumor material

In order to study genetic aspects of EAC development, intercross (F1, F2) and backcross (N1) populations were set up by breeding BDII females to males from two different strains with low EAC incidence (BN and SPRD*Cu3*). Females were examined weekly, suspected tumors were were surgically removed and pathologically examined, and cell cultures were established when possible [24]. The tumors were pathologically classified as EAC, or other uterine tumors. In some cases no cancer cells were detected when pathologically examined. These tissues were referred to non-malignant endometrium (NME) (Table 1) [8,30]. In this study cell lines from tumors and pre/nonmalignant tissues in the backcross female progeny were used (NUT).

### *In vitro* cell culture conditions

Primary cell cultures from endometrial material (EAC and NME) and rat embryo fibroblast (REF) cell line were cultured in Dulbecco’s modified Eagle medium (DMEM, Invitrogen) supplemented with 100 IU/100 μg/ml penicillin/streptomycin, L-glutamine, MEM amino acids, MEM Non-Essential Amino acids, MEM Vitamins solution and 10% heat-inactivated fetal bovine serum, for 3–5 passages in order to obtain the required amount of cells. The cells were grown at 37°C with a 5% CO_2_ and humidity at 95% and harvested by trypzination at a confluence of 80-90%. In this study, a total of 22 primary cell cultures (11 EAC and 11 NME) were used (Table 1).

### Human tumor material

A total of 30 EACs in FIGO grade I-III (10 tumors from each grade) embedded in archival formalin fixed paraffin (FFPE) were used in the study. As control, 26 benign FFPE endometrial tissues (12 of secretory phase and 14 of proliferative phase) were used. As the reference sample, one sample from normal endometrium was used in the normalization process (Table 2) [31] for further details).

### Exctraction av RNA and RT-PCR

Total RNA was isolated from the EAC/NME cell lines (Table 1) using the Qiagen® AllPrep RNA Mini Kit according to the manufacturer’s protocol. Total RNA was extracted from human endometrial paraffin imbedded tissue samples (Table 2) using Qiagen® AllPrep RNA FFPE Kit. RT-PCR was performed on 500 ng of total RNA, using the High Capacity RNA-to-cDNA Kit according to the manufacturer’s protocol (Applied Bio-systems, USA).

### Quantitative PCR (qPCR) of *Phf5a* and *Gja1* in rat tumors

A total of 11 EAC and 11 NME cell lines were included in the qPCR analysis. The house keeping gene, *Gapdh* was used as an endogenous control and the Rat embryo fibroblast cell line (REF) was used as an exogenous control. Template cDNA was added to TaqMan Universal Master Mix (Applied Biosystems, USA) in a 25 μl reaction with specific pre-designed probes for *Phf5,a Gja1 and Gapdh* (Applied Biosystems). Reactions were performed in triplicates and the averages threshold cycle number was used for further analysis. Relative gene expression quantification was calculated according to the comparative Ct method using *Gapdh* as an endogenous control and REF as calibrator. Final results were determined as follows: 2^–(ΔCt sample–ΔCt calibrator)^, where ΔCt values of the calibrator and sample were determined by subtracting the Ct value of the target gene from the value of the *Gapdh* gene.

### Quantitative PCR (qPCR) of *PHF5A* and *GJA1* in human tumors

Total RNA from paraffin imbedded tissue samples was used for qPCR. The house keeping gene, *GAPDH* was used as an endogenous control and RNA from the endometrial tissue was used as an exogenous control. The qPCR reaction setup followed the same procedure as for the rat samples.

### Statistical analysis

For statistical evaluations of gene expression data, Ct values for differences among replicates was analyzed by ANOVA. For comparisons of expression differences between normal and malignant tissues, independent sample *t*-test was applied on the log 2-fold change (PASW Statistics 20, SPSS Inc, Chicago, USA). In both tests the null hypotheses were assuming no differences between replicates, and no differences between tissue types, respectively. The Pearson correlation test was performed to check for expression correlation between *Phf5a/PHF5A* and *Gja1/ GJA1*. The significance levels were set to P<0.05 in all statistical tests. Furthermore, the tumor material from the rat samples was analyzed for differences in gene expression between the two backgrounds (BN and SPRD).

## Abbreviations

EAC: Endometrial adenocarcinoma; NME: Normal/pre-malignant endometrium; NUT: Backcross, rat uterine tumor; FIGO: International Federation of Gynecology and Obstetrics.

## Competing interests

The authors declare that they have no competing interests.

## Authors’ contributions

EF contributed with ides, designed the study, performed all the experiment, data analysis and drafted the manuscript. KKL supervised the project, contributed with ideas and took part in the preparation of the manuscript. Both authors have read and approved the final version of the manuscript.
